# Supplementary material for: Mosaic gastruloids reveal a temporal restriction for developmental cell competition
Source: Nat Cell Biol. 2026 Apr 1;28(5):875–89. doi: 10.1038/s41556-026-01923-x (PMC13179131; doi:10.1038/s41556-026-01923-x)
Supplement: Supplementary file 1 — Supplementary Figs. 1–3 and supplementary figure legends. [file 41556_2026_1923_MOESM1_ESM.pdf]

nature cell biology

Supplementary information https://doi.org/10.1038/s41556-026-01923-x

Mosaic gastruloids reveal a temporal restriction for developmental cell competition

In the format provided by the authors and unedited

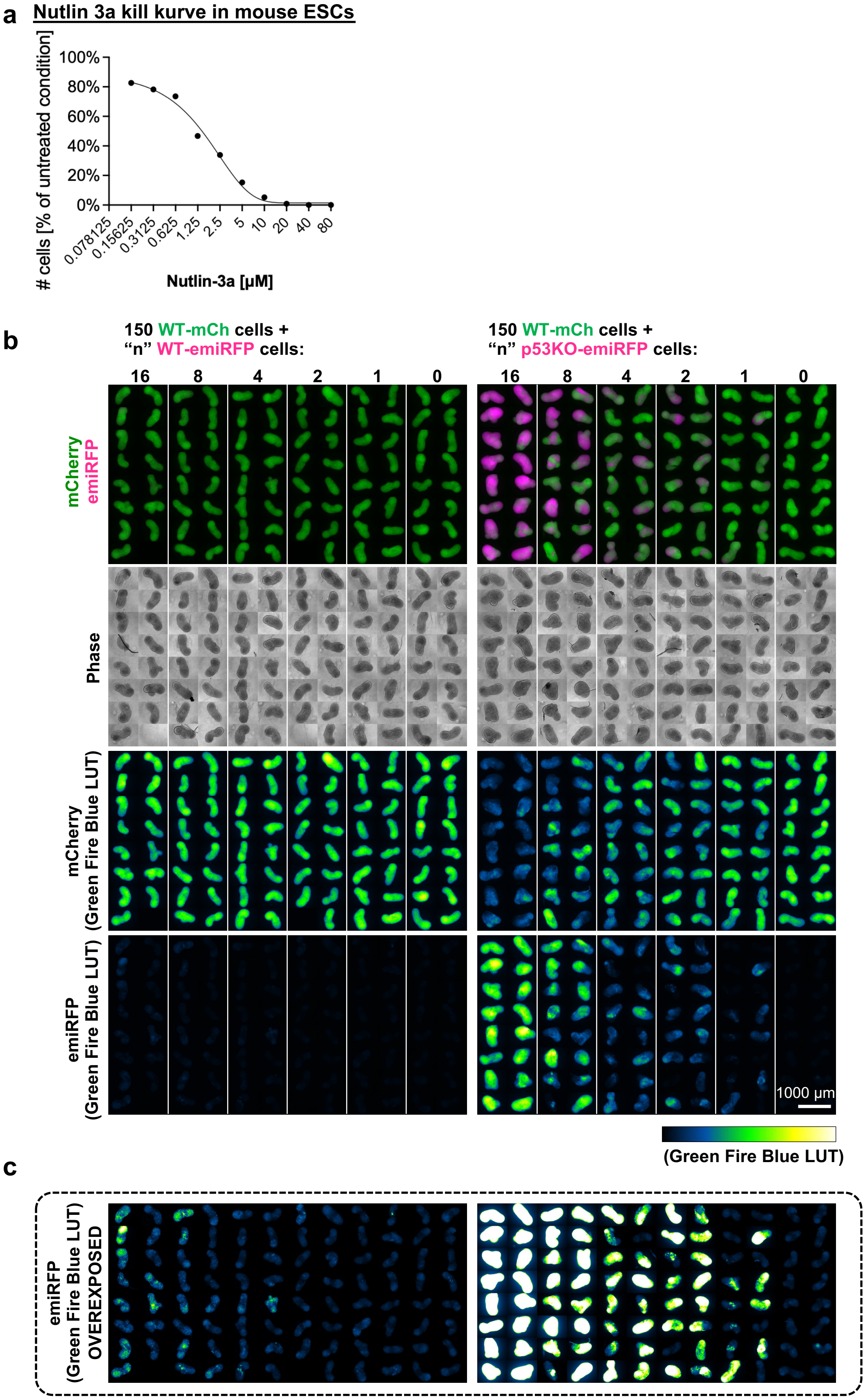

a
Nutlin 3a kill kurve in mouse ESCs
cells [% of untreated condition]
100%
80%
60%
40%
20%
0%
0.078125
0.15625
0.3125
0.625
1.25
2.5
5
10
20
40
80
Nutlin-3a [µM]
b
150 WT-mCh cells +
"n" WT-emiRFP cells:
150 WT-mCh cells +
"n" p53KO-emiRFP cells:
16
8
4
2
1
0
mCherry
emiRFP
Phase
mCherry
(Green Fire Blue LUT)
emiRFP
(Green Fire Blue LUT)
1000 µm
(Green Fire Blue LUT)
c
emiRFP
(Green Fire Blue LUT)
OVEREXPOSED

Supplementary Figure 1.

a, Nutlin-3a dose-response kill curve showing viability of WT E14Tg2A mESC cells after 96h treatment with varying Nutlin-3a concentrations, expressed as percent viable cells of untreated control.

b, Corresponds to Fig. 1f. Cell number titration of 0-16 WT-emiRFP (left) or p53KO-emiRFP (right) cells seeded in mosaic gastruloids with 150 WT-mCherry cells each. Representative widefield fluorescence microscopy of 120h gastruloids. Montages depicted as merged mCherry+emiRFP channels, phase contrast, and single channels displayed with a Green-Fire-Blue look-up-table (LUT).

c, Purposely overexposed emiRFP channel in Green-Fire-Blue LUT to visualize rare emiRFP cells at low seeding numbers.

Scale bar denotes 1,000 μm.

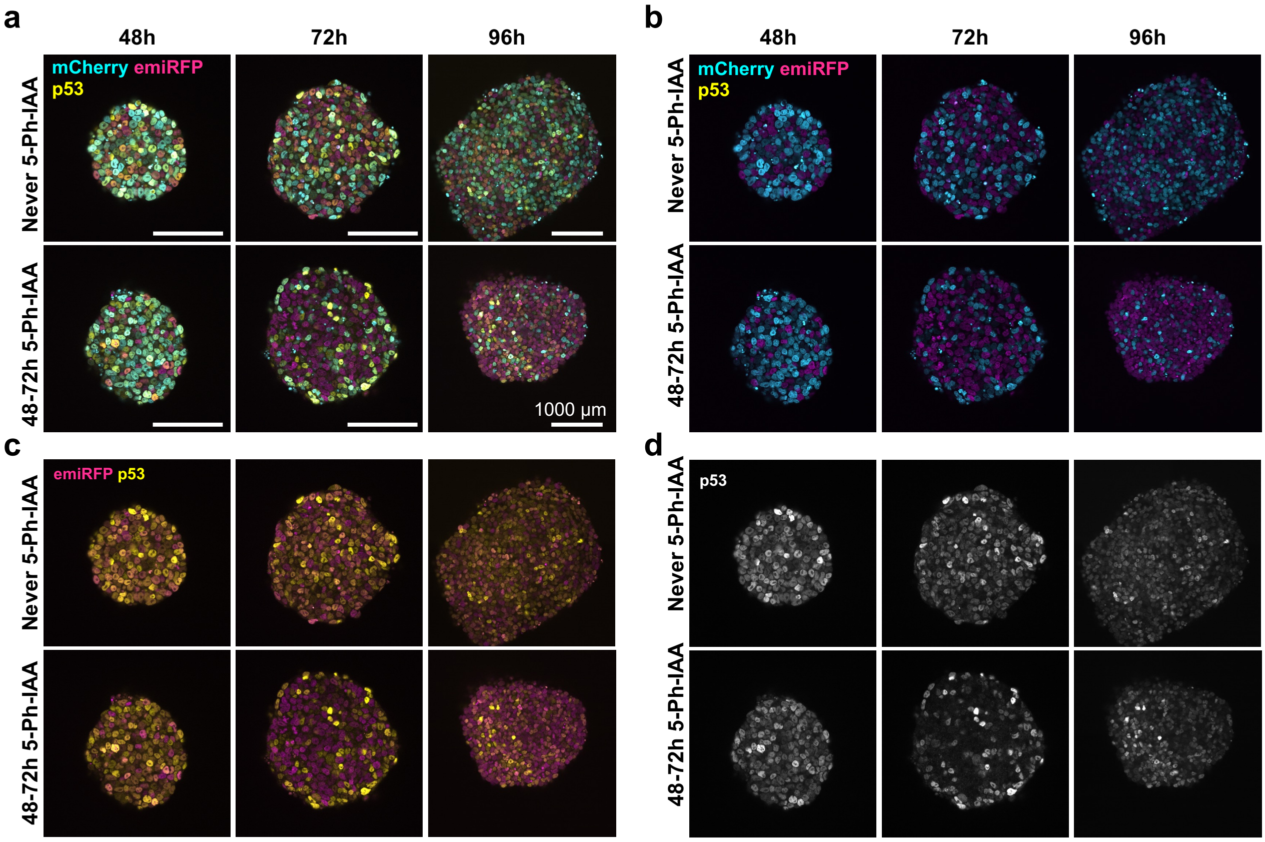

a
48h
72h
96h
mCherry emiRFP p53
Never 5-Ph-IAA
48-72h 5-Ph-IAA
1000 µm
b
48h
72h
96h
mCherry emiRFP p53
Never 5-Ph-IAA
48-72h 5-Ph-IAA
c
emiRFP p53
Never 5-Ph-IAA
48-72h 5-Ph-IAA
d
p53
Never 5-Ph-IAA
48-72h 5-Ph-IAA

Supplementary Figure 2. Single and double channel overlay images of degron gastruloids.
a-d, Representative confocal images of p53-immunostained WT+p53degron gastruloids at indicated times (top label) in presence or absence of 100 nM 5-Ph-IAA treatment during 48-72h after aggregation. Merged overlays of all channels (**a**), of mCherry (cyan) with emiRFP (magenta) (**b**), of emiRFP with p53 (yellow) (**c**), or single gray scale p53 channel (**d**) displayed. Overlay of emiRFP and p53 channels (**c**) serves to demonstrate absence of double positive nuclei at 72h after 5-Ph-IAA treatment. Corresponds to Fig. 8h.
Scale bars denote 1,000 μm.

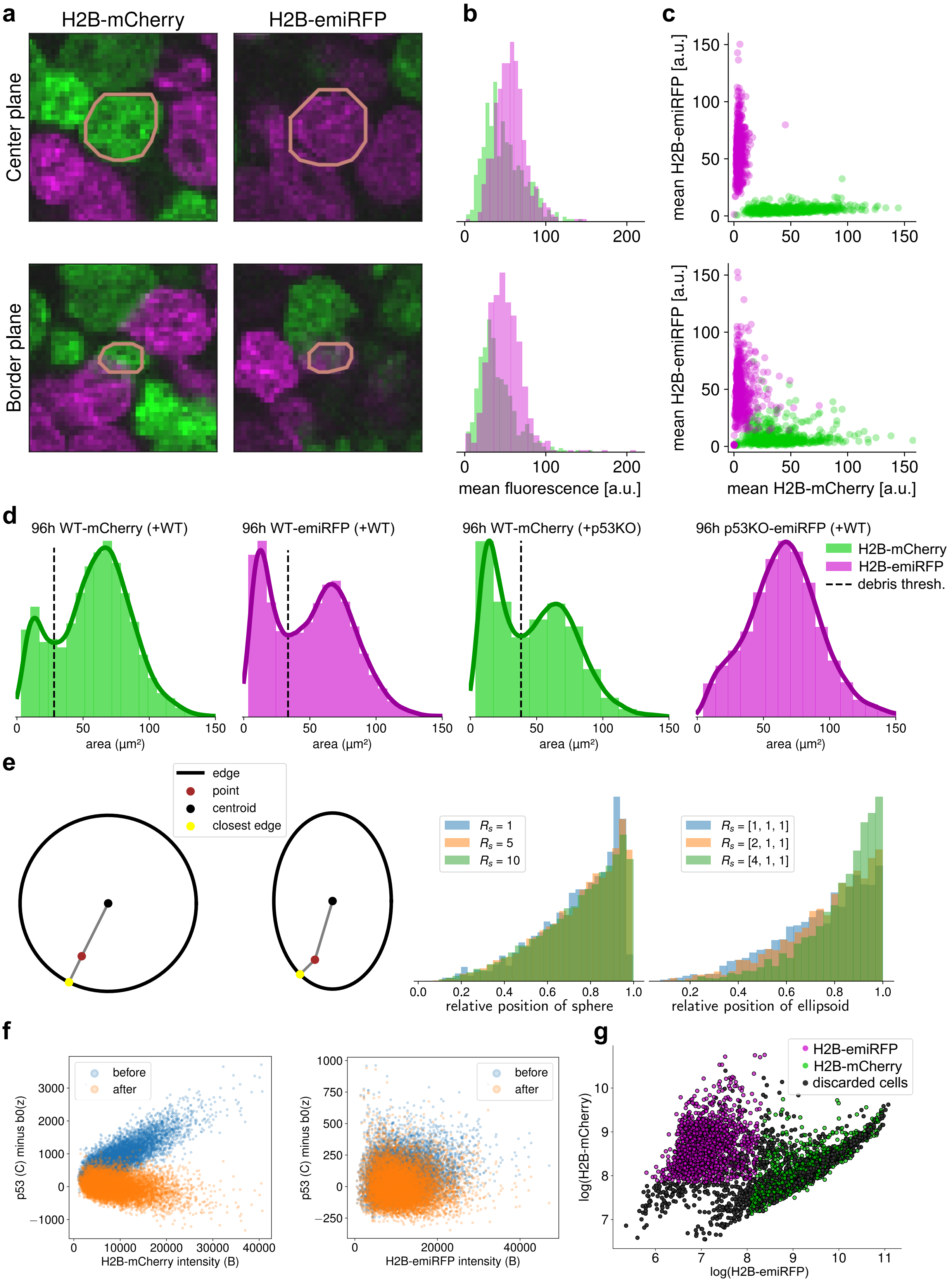

a
H2B-mCherry
H2B-emiRFP
Center plane
Border plane
b
mean fluorescence [a.u.]
c
mean H2B-emiRFP [a.u.]
mean H2B-mCherry [a.u.]
d
96h WT-mCherry (+WT)
96h WT-emiRFP (+WT)
96h WT-mCherry (+p53KO)
96h p53KO-emiRFP (+WT)
H2B-mCherry
H2B-emiRFP
debris thresh.
area (μm²)
e
edge
point
centroid
closest edge
R_s = 1
R_s = 5
R_s = 10
R_s = [1, 1, 1]
R_s = [2, 1, 1]
R_s = [4, 1, 1]
relative position of sphere
relative position of ellipsoid
f
before
after
p53 (C) minus b0(z)
H2B-mCherry intensity (B)
H2B-emiRFP intensity (B)
g
H2B-emiRFP
H2B-mCherry
discarded cells
log(H2B-mCherry)
log(H2B-emiRFP)

Supplementary Figure 3. Image analysis pipeline quality controls and details.

a, Examples of WT-mCherry (left) and WT-emiRFP (right) segmented nuclei at their center (top) or edge (bottom) plane. Brown outline denotes the image analysis detected nuclear region.

b, Fluorescence quantification of the segmented channel for each of the populations.

c, Visualization of signal overlap at the center (top) and the edge (bottom) of the nuclei. Conversely to panel (b), here we quantify both channels for each segmented cell.

d, Size (area) distributions of segmented nuclei from 96h gastruloids for different experimental conditions. The vertical dash line represents the threshold between debris and true cells. p53KO cells do not have an apparent debris peak.

e, Schematic representation of the sphere and ellipsoid tests in 2d (left) and distributions of relative distances for spheres and ellipsoids (right).

f, Example of spillover correction between the p53 channel and the mCherry (left) or the emiRFP (right).

g, Example of H2B-mCherry vs. H2B-emiRFP misclassification.
