# Supplementary material for: Mosaic gastruloids reveal a temporal restriction for developmental cell competition
Source: Nat Cell Biol. 2026 Apr 1;28(5):875–89. doi: 10.1038/s41556-026-01923-x (PMC13179131; doi:10.1038/s41556-026-01923-x)
Supplement: Supplementary file 2 — Reporting Summary [file 41556_2026_1923_MOESM2_ESM.pdf]

nature portfolio

Corresponding author(s): Joshua D Frenster, Alfonso Martinez Arias

Last updated by author(s): Jan 18, 2026

Reporting Summary

Nature Portfolio wishes to improve the reproducibility of the work that we publish. This form provides structure for consistency and transparency in reporting. For further information on Nature Portfolio policies, see our Editorial Policies and the Editorial Policy Checklist.

Statistics

For all statistical analyses, confirm that the following items are present in the figure legend, table legend, main text, or Methods section.

n/a	Confirmed	
☐	☒	The exact sample size (*n*) for each experimental group/condition, given as a discrete number and unit of measurement
☐	☒	A statement on whether measurements were taken from distinct samples or whether the same sample was measured repeatedly
☐	☒	The statistical test(s) used AND whether they are one- or two-sided<br>*Only common tests should be described solely by name; describe more complex techniques in the Methods section.*
☐	☒	A description of all covariates tested
☐	☒	A description of any assumptions or corrections, such as tests of normality and adjustment for multiple comparisons
☐	☒	A full description of the statistical parameters including central tendency (e.g. means) or other basic estimates (e.g. regression coefficient) AND variation (e.g. standard deviation) or associated estimates of uncertainty (e.g. confidence intervals)
☐	☒	For null hypothesis testing, the test statistic (e.g. *F*, *t*, *r*) with confidence intervals, effect sizes, degrees of freedom and *P* value noted<br>*Give P values as exact values whenever suitable.*
☒	☐	For Bayesian analysis, information on the choice of priors and Markov chain Monte Carlo settings
☐	☒	For hierarchical and complex designs, identification of the appropriate level for tests and full reporting of outcomes
☒	☐	Estimates of effect sizes (e.g. Cohen's *d*, Pearson's *r*), indicating how they were calculated

Our web collection on statistics for biologists contains articles on many of the points above.

Software and code

Policy information about availability of computer code

Data collection	FlowJo (version 10.10.0), BD FACS Diva (version 6.2), Zeiss ZEN (version 3.11), Zeiss ZEN Blue (version 3.2), Harmony PhenoLOGIC (version 5.2), EPview (version v2.9.22), FastQC (version 0.11.9), TrimGalore (version 0.6.7), STAR aligner (version 2.7.11b)
Data analysis	ImageJ2 (version 2.14.0/1.54f), GraphPad Prism (version 10.4.1(532)); RNAseq analysis: Python (version 3.12.8), PyDESeq2 (version 0.4.0), scikit-learn library (version 1.2.2), seaborn (version 0.13.2), matplotlib (version 3.9.2); Image analysis: Python (version 3.9.13), qlivecell (version 0.7 available at https://github.com/dsb-lab/qlivecell), GSEA (version 4.3.3)<br><br>Custom code available under: https://github.com/stembryo-lab/cell_competition_gastruloids

For manuscripts utilizing custom algorithms or software that are central to the research but not yet described in published literature, software must be made available to editors and reviewers. We strongly encourage code deposition in a community repository (e.g. GitHub). See the Nature Portfolio guidelines for submitting code & software for further information.

Data

Policy information about availability of data

All manuscripts must include a data availability statement. This statement should provide the following information, where applicable:

- Accession codes, unique identifiers, or web links for publicly available datasets
- A description of any restrictions on data availability
- For clinical datasets or third party data, please ensure that the statement adheres to our policy

All RNA sequencing datasets generated in this study have been deposited and are publicly available in the Gene Expression Omnibus under accession no. GSE294530.

Research involving human participants, their data, or biological material

Policy information about studies with human participants or human data. See also policy information about sex, gender (identity/presentation), and sexual orientation and race, ethnicity and racism.

Reporting on sex and gender	N/A
Reporting on race, ethnicity, or other socially relevant groupings	N/A
Population characteristics	N/A
Recruitment	N/A
Ethics oversight	N/A

Note that full information on the approval of the study protocol must also be provided in the manuscript.

Field-specific reporting

Please select the one below that is the best fit for your research. If you are not sure, read the appropriate sections before making your selection.

☒ Life sciences ☐ Behavioural & social sciences ☐ Ecological, evolutionary & environmental sciences

For a reference copy of the document with all sections, see nature.com/documents/nr-reporting-summary-flat.pdf

Life sciences study design

All studies must disclose on these points even when the disclosure is negative.

Sample size	No statistical method were used to predetermine the samples sizes, but each result was repeated in at least 3 independent experiments, containing several technical repeats per biological repeat. Numbers of independent repeats and number of gastruloids per repeat are annotated in each figure legend.
Data exclusions	Individual gastruloids were only excluded from quantitative analysis of cell number counts if their counts were unreliable due to technical problems like loss of cells during the handling procedure of dissociation. No other data was excluded.
Replication	All experiments were replicated in at least 3 independent experiments. RNAseq samples were collected from 3 temporally separate independent experiments. Within each independent experiment multiple gastruloids were analyzed.
Randomization	Gastruloids were not specifically chosen for any experiment and individual gastruloids for quantification were picked randomly from 96 well plates without prior analysis or preference to specific gastruloids. Sequencing data was analyzed without human intervention in an unbiased fashion.
Blinding	Data collection and analysis were not performed blind to the conditions of the experiments. Blinding was not required for this study since it is not an intervention study and does not contain subjective or qualitative assessment. All samples were analyzed using the same uniform parameters in an unbiased manner.

Reporting for specific materials, systems and methods

We require information from authors about some types of materials, experimental systems and methods used in many studies. Here, indicate whether each material, system or method listed is relevant to your study. If you are not sure if a list item applies to your research, read the appropriate section before selecting a response.

Materials & experimental systems

n/a	Involved in the study
☐	☒ Antibodies
☐	☒ Eukaryotic cell lines
☒	☐ Palaeontology and archaeology
☒	☐ Animals and other organisms
☒	☐ Clinical data
☒	☐ Dual use research of concern
☒	☐ Plants

Methods

n/a	Involved in the study
☒	☐ ChIP-seq
☐	☒ Flow cytometry
☒	☐ MRI-based neuroimaging

Antibodies

Antibodies used

Primary antibodies:
p53 (WB) (Leica, Cat# NCL-L-p53-CM5p, 1:1000), p53(IF, Flow)(Cell Signaling, Cat# 2524S, clone 1C12, 1:4,000), beta-actin (Santa Cruz, Cat# sc-47778, 1:2000), E-Cadherin (R&D, Cat# AF648, 1:1000), N-Cadherin (Abcam, Cat# ab18203, 1:200), FoxA2 (Cell Signaling, Cat# 8186, 1:400), Tbx6 (R&D, Cat# AF4744, 1:200), T/Brachyury (R&D, Cat# AF2085, 1:100), Sox2 (Abcam, Cat# ab92494, 1:200), Otx2 (R&D, Cat# AF1979, 1:200), Sox3 (ThermoFisher, Cat# PA5-35983, 1:300), Nanog (Invitrogen, Cat# 14-5761-80, 1:250), phospho-Histone3 (Cell Signaling, Cat# 3377, 1:1600), cleaved Caspase3 (Cell Signalling, Cat# 9664, 1:500), YAP (Cell Signaling, Cat# 14074, clone D8H1X, 1:200), CD31/PECAM-1 AlexaFluor 488 conjugated (R&D, Cat# FAB3628G-025, 1:400), SSEA-1 AlexaFluor 405 conjugated (R&D, Cat# FAB2155V-100UG, 1:400).

Secondary antibodies: HRP-conjugated mouse anti-rabbit (Santa Cruz, Cat# sc2357, 1:2000), HRP-conjugated m-IgGk BP anti-mouse (Santa Cruz, Cat# sc-516102, 1:2000), Alexa Fluor 488 Donkey anti-Goat (Invitrogen, Cat# A-11055, 1:500), Alexa Fluor 488 Goat anti-Rabbit (Invitrogen, Cat# A-11034, 1:500), Alexa Fluor 488 Goat anti-Rat (Invitrogen, Cat# A-11006, 1:500).

Validation

Antibodies were validated by their manufacturers:
p53 antibody for Western blot: https://shop.leicabiosystems.com/ihc-ish/ihc-primary-antibodies/pid-p53-protein-cm5
p53 antibody for Immunofluorescence and Flow cytometry: https://www.cellsignal.com/products/primary-antibodies/p53-1c12-mouse-monoclonal-antibody/2524
beta-actin atibody: https://www.scbt.com/p/beta-actin-antibody-c4
E-Cadherin antibody: https://www.rndsystems.com/products/human-mouse-e-cadherin-antibody_af648
N-Cadherin antibody: https://www.abcam.com/en-us/products/primary-antibodies/n-cadherin-antibody-intercellular-junction-marker-ab18203
FoxA2 antibody: https://www.cellsignal.com/products/primary-antibodies/foxa2-hnf3b-d56d6-xp-rabbit-mab/8186
Tbx6 antibody: https://www.rndsystems.com/products/human-tbx6-antibody_af4744
Brachyury abntibody: https://www.rndsystems.com/products/human-mouse-brachyury-antibody_af2085
Sox2 antibody: https://www.abcam.com/en-us/products/primary-antibodies/sox2-antibody-epr3131-ab92494
Otx2 antibody: https://www.rndsystems.com/products/human-otx2-antibody_af1979
Sox3 antibody: https://www.thermofisher.com/antibody/product/SOX3-Antibody-Polyclonal/PA5-35983
Nanog antibody: https://www.thermofisher.com/antibody/product/Nanog-Antibody-clone-eBioMLC-51-Monoclonal/14-5761-80
Phospho-Histone3 antibody: https://www.cellsignal.com/products/primary-antibodies/phospho-histone-h3-ser10-d2c8-xp-rabbit-mab/3377
Cleaved Caspase 3 antibody: https://www.cellsignal.com/products/primary-antibodies/cleaved-caspase-3-asp175-5a1e-rabbit-mab/9664
YAP: https://www.cellsignal.com/products/primary-antibodies/yap-d8h1x-rabbit-monoclonal-antibody/14074
PECAM-1 Alexa Fluor 488-conjugated antibody: https://www.rndsystems.com/products/mouse-rat-cd31-pecam-1-alexa-fluor-488-conjugated-antibody_fab3628g
SSEA-1 Alexa Fluor 405-conjugated antibody: https://www.rndsystems.com/products/human-mouse-ssea-1-alexa-fluor-405-conjugated-antibody-mc-480_fab2155v
Donkey anti-Goat IgG Alexa Fluor 488 conjugated antibody: https://www.thermofisher.com/antibody/product/Donkey-anti-Goat-IgG-H-L-Cross-Adsorbed-Secondary-Antibody-Polyclonal/A-11055
Goat anti-Rabbit IgG Alexa Fluor 488 conjugated antibody: https://www.thermofisher.com/antibody/product/Goat-anti-Rabbit-IgG-H-L-Highly-Cross-Adsorbed-Secondary-Antibody-Polyclonal/A-11034
Goat anti-Rat IgG Alexa Fluor 488 conjugated antibody: https://www.thermofisher.com/antibody/product/Goat-anti-Rat-IgG-H-L-Cross-Adsorbed-Secondary-Antibody-Polyclonal/A-11006
HRP-conjugated m-IgGk BP anti-mouse: https://www.scbt.com/p/m-igg-kappa-bp-hrp
HRP-conjugated mouse anti-rabbit : https://www.scbt.com/p/mouse-anti-rabbit-igg-hrp

Eukaryotic cell lines

Policy information about cell lines and Sex and Gender in Research

Cell line source(s)

All experiments of this manuscript were conducted in the E14TG2a mouse embryonic stem cell line (https://www.atcc.org/products/crl-1821) derived by T Doetschman from 129/Ola mice in 1987. All mutant clones were generated from this parental line in house, with the exception of Brachyury-knockout, Eomesodermin-knockout, and Bra/Eomes-double-knockout cells, which were derived in the laboratory of Dr. Sebastian Arnold, University of Freiburg.

Authentication

None of the cell lines were authenticated.

Mycoplasma contamination

All cell lines used in this study were regularly tested negative for mycoplasma.

Commonly misidentified lines (See ICLAC register)

No commonly misidentified cell lines were used in this study.

Plants

Seed stocks

N/A

Novel plant genotypes

N/A

Authentication

N/A

Flow Cytometry

Plots

Confirm that:

☒ The axis labels state the marker and fluorochrome used (e.g. CD4-FITC).

☒ The axis scales are clearly visible. Include numbers along axes only for bottom left plot of group (a 'group' is an analysis of identical markers).

☒ All plots are contour plots with outliers or pseudocolor plots.

☒ A numerical value for number of cells or percentage (with statistics) is provided.

Methodology

Sample preparation

Gastruloids were collected at indicated timepoints, washed in PBS-/-, and enzymatically dissociated using Accutase (Lab Clinics Capricorn Scientific, Cat# ACC-1B). Optimal dissociation was achieved by warming tubes containing gastruloids and Accutase to 37C in a water bath while shaking and eventually flicking the tubes during a time course of 5 minutes. Gastruloids were further dissociated mechanically using a p1000 pipette while adding flow buffer (PBS-/-, 2% BSA, 2mM EDTA). Resulting single cell suspensions were washed in flow buffer and pelleted by centrifugation at 200 xg for 3 minutes. Single gastruloid flow as depicted in Figure 2 of this manuscript was conducted by analyzing the dissociated gastruloids live at this step of the protocol.
For antibody stained flowcytometry multiple gastruloids of the same time point and batch were pooled and stained together. Single cells were washed one more time in PBS-/- followed by fixation in 4% PFA for 15 minutes. Fixed cells were washed 3 times in flow buffer, followed by blocking in 10% BSA in PBS-/- containing 0.1% Triton X-100 for 30 minutes at room temperature. After setting aside a fraction of the sample for use as negative or secondary antibody control, primary antibodies were diluted in 2% BSA in PBS-/- with 0.1%Triton X-100 (staining buffer). Cells were incubated with primary antibodies for 1 hour shaking at room temperature, followed by 3 washing steps in staining buffer. Fluorophore-conjugated secondary antibodies were diluted 1:500 in staining buffer unless otherwise stated and used to stain cells for 30 minutes at room temperature shaking. Cells were washed 3 times in staining buffer again and transferred to flow cytometry tubes for analysis.

Instrument

All samples were analyzed using a BD Bioscience LSRFortessa system.

Software

Analysis was conducted using: FlowJo (version 10.10.0) and BD FACS Diva (version 6.2)

Cell population abundance

Flow cytometry was used primarily as final readout and not to generate input of experimental starting material, with the exception of RNAseq samples isolated from mosaic gastruloids. Due to the temporal contraints of analysing sorted cells quickly before loss or change of RNAquality, purity of these samples was not validated after FACS isolation.

Gating strategy

All flow cytometry analysis started with pregating of cells vs debris using FSC-A vs SSC-A channels. This was followed by an exclusion of doublets using FSC-A vs FSC-H and SSC-A vs SSC-H. For population percentages, mCherry-positive and emiRFP670-positive events were then quantified. For phosho-Histone 3, SSEA-1, Pecam-1 staining, cells were pre-gated for mCherry or emiRFP670 positivity, and then analyzed separately in their respective stained channels.

☒ Tick this box to confirm that a figure exemplifying the gating strategy is provided in the Supplementary Information.
