# Supplementary material for: Mosaic gastruloids reveal a temporal restriction for developmental cell competition
Source: Nat Cell Biol. 2026 Apr 1;28(5):875–89. doi: 10.1038/s41556-026-01923-x (PMC13179131; doi:10.1038/s41556-026-01923-x)
Supplement: Supplementary file 22 — Unprocessed western blots from Figs. 1 and 8 and Extended Data Fig. 1. Clearly labelled, together in one file. [file 41556_2026_1923_MOESM22_ESM.pdf]

Unprocessed membranes of Figure 1a and Extended Data Figure 1d

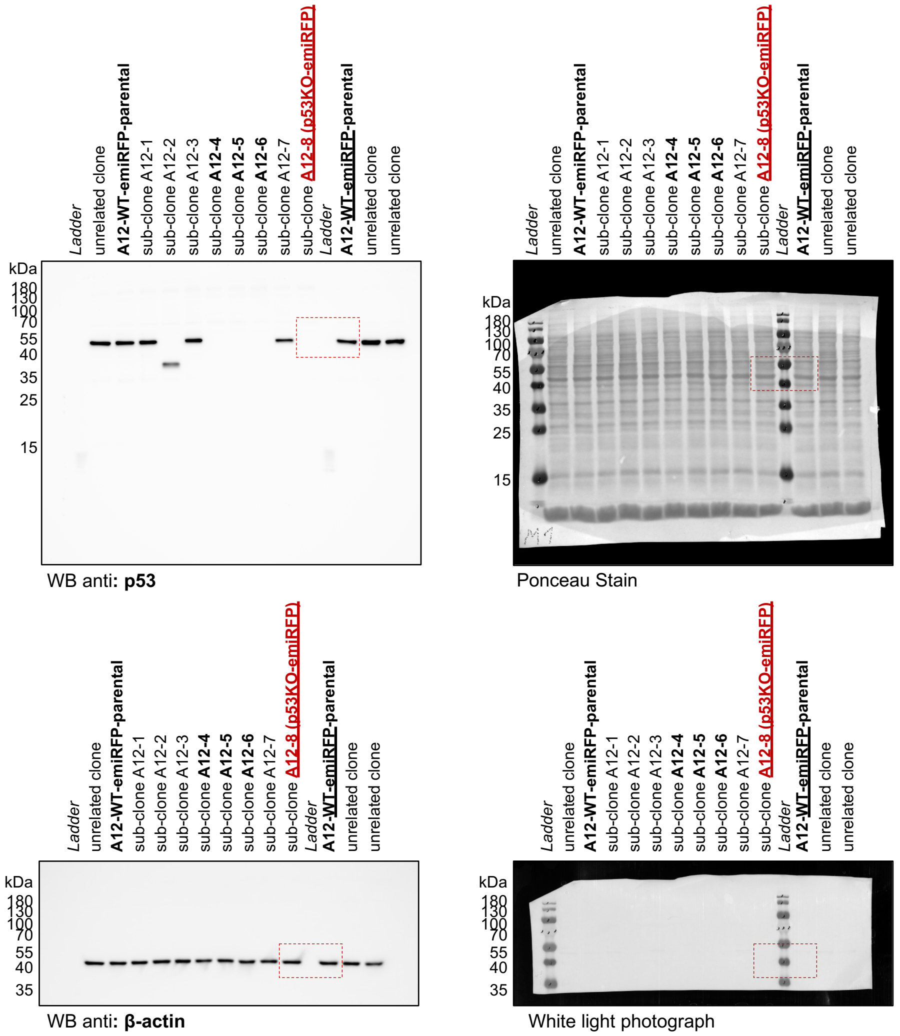

Red dashed box indicates the cropped region depicted in Fig. 1a. The same membrane was stained against p53 and the loading control β-actin sequentially. The same membranes are depicted under white light (right) and as exposed chemiluminescence (left).

Unprocessed membranes of Figure 8a

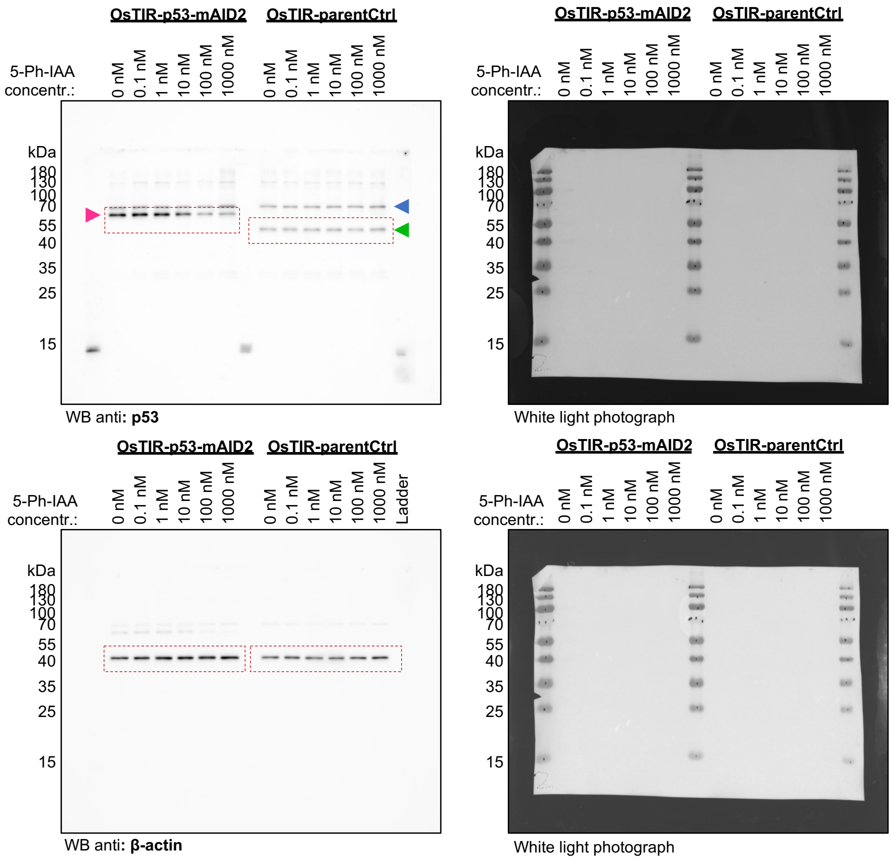

Unprocessed membranes of Figure 8e

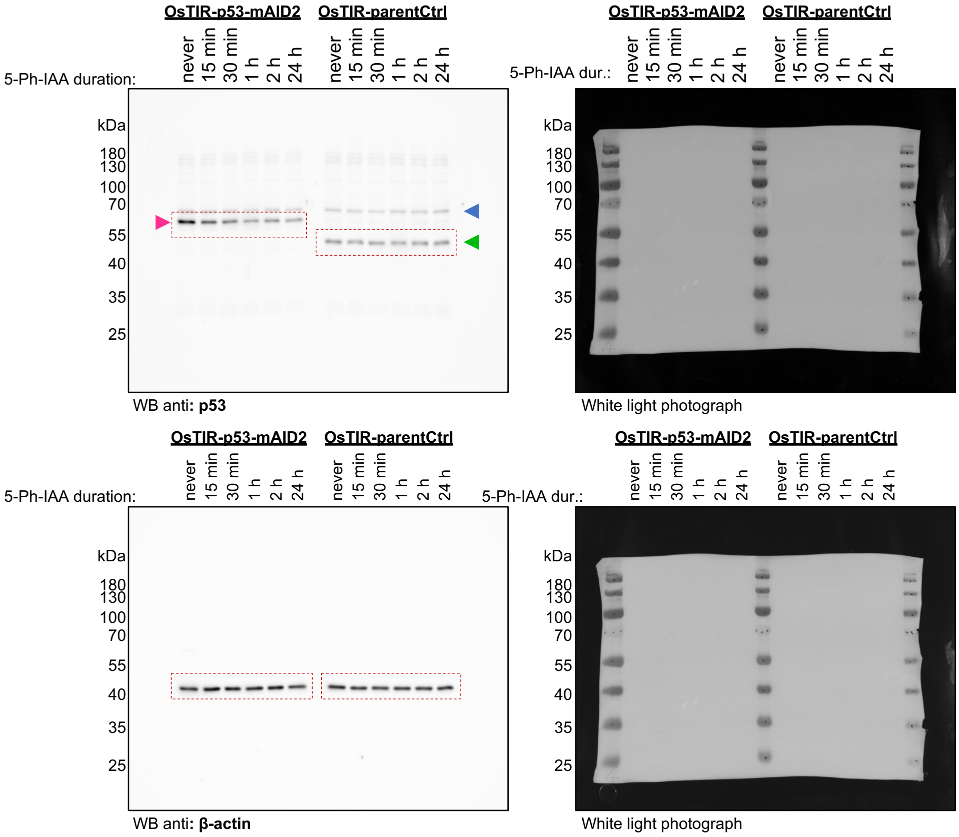

Red dashed box indicates the cropped regions depicted in Fig. 8a and Fig. 8e. The same membranes were stained against p53 and the loading control β-actin sequentially. The same membranes are depicted under white light (right) and as exposed chemiluminescence (left). Magenta arrowheads mark p53-mAID2 with size shift due to the degron tag. Green arrowheads mark wildtype p53 as detected in the OsTIR-parentCtrl clone that does not have a mAID2 tag in the genome. Blue arrowheads mark an off-target band that is unchanged in all conditions.
